# The Eastern Nebraska Salt Marsh Microbiome Is Well Adapted to an Alkaline and Extreme Saline Environment

**DOI:** 10.3390/life11050446

**Published:** 2021-05-15

**Authors:** Sierra R. Athen, Shivangi Dubey, John A. Kyndt

**Affiliations:** College of Science and Technology, Bellevue University, Bellevue, NE 68005, USA; sathen@my365.bellevue.edu (S.R.A.); shdubey@bellevue.edu (S.D.)

**Keywords:** salt marsh, microbiome, *Rubribacterium*, *Prosthecochloris*, *Marichromatium*, *Sulfurimonas*, *Sulfurospirillum*, sulfur cycling, Salt Creek

## Abstract

The Eastern Nebraska Salt Marshes contain a unique, alkaline, and saline wetland area that is a remnant of prehistoric oceans that once covered this area. The microbial composition of these salt marshes, identified by metagenomic sequencing, appears to be different from well-studied coastal salt marshes as it contains bacterial genera that have only been found in cold-adapted, alkaline, saline environments. For example, *Rubribacterium* was only isolated before from an Eastern Siberian soda lake, but appears to be one of the most abundant bacteria present at the time of sampling of the Eastern Nebraska Salt Marshes. Further enrichment, followed by genome sequencing and metagenomic binning, revealed the presence of several halophilic, alkalophilic bacteria that play important roles in sulfur and carbon cycling, as well as in nitrogen fixation within this ecosystem. Photosynthetic sulfur bacteria, belonging to *Prosthecochloris* and *Marichromatium*, and chemotrophic sulfur bacteria of the genera *Sulfurimonas*, *Arcobacter*, and *Thiomicrospira* produce valuable oxidized sulfur compounds for algal and plant growth, while alkaliphilic, sulfur-reducing bacteria belonging to *Sulfurospirillum* help balance the sulfur cycle. This metagenome-based study provides a baseline to understand the complex, but balanced, syntrophic microbial interactions that occur in this unique inland salt marsh environment.

## 1. Introduction

Over 100 million years ago, Nebraska was covered by a vast sea. Now, all that is left of that are widespread salt marshes, which makes this one of the few places in the U.S. where the naturally occurring groundwater is saline [1]. The alkaline and saline marshes in Nebraska are part of a rare wetland type that occurs in the western Sandhills, the North Platte Valley, and the valley of Salt Creek and Little Salt Creek [2]. In these areas, the salinity is so high that it creates a unique natural community in the middle of the tallgrass prairie that is selective to the growth of only salt-adapted species. For example, the Salt Creek tiger beetle cannot be found anywhere else on Earth [3,4], and Saltwort (*Salicornia rubra*) is a state-listed endangered plant species that, in Nebraska, is only found in these saline wetlands [5,6].

However, urban expansion and consequential changes to the hydrological systems have endangered the continuing existence of these unique salt marshes. Only about 4000 acres remain scattered throughout the region of the estimated 20,000 acres that once existed [1].

**Figure 1:**
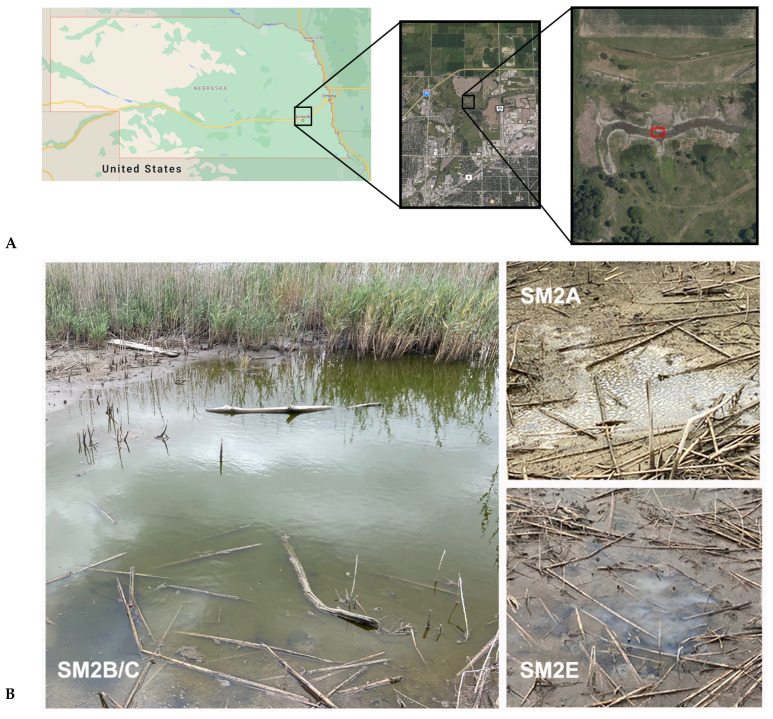
(**A**) Map of the sampling locations for this study. Samples were taken at the valley of Salt Creek and Little Salt Creek near Lincoln, Nebraska. The sampling area within the salt marsh is marked in red. All maps were generated using Google Maps (**B**) Overview of the NE Salt Marsh sampling locations: SM2B, SM2C, SM2A, and SM2E.

In 2003, the Nature Conservancy helped form the Saline Wetlands Conservation Partnership as a way to work collaboratively among organizations concerned with preserving the last remnants of Nebraska’s saline wetlands. One of the ongoing scientific efforts is to understand exactly how these wetlands became salty, besides studying the larger ecological impact of these unique habitats. In particular, the smaller salt marsh area of the Salt Creek valley, near Lincoln, NE, is the most endangered by growing urban development. Most of the nearby surrounding area of that lower Salt Creek valley area has been used for the development of roads, industrial and residential properties, or agricultural expansion (Figure 1A). The Marsh Wren saline wetland restoration project was started to conserve and restore approximately 150 acres containing saline wetlands and other habitats of the lower Salt Creek valley and was completed in 2017 [7]. A combination of traditional restoration methods and physical manipulation of hydrology through pumping of saline groundwater to the wetland surface was used.

The bacterial composition of coastal salt marshes has been well studied on a global scale [8,9,10]. On the contrary, these Nebraska salt marshes are thousands of miles away from any coast and have not been part of a larger saline body of water for apparently millions of years. Very little is known about the microbial composition of these salt marshes, how this compares to other coastal or marine environments, or how the bacterial species and their metabolism potentially impact the rest of the local ecosystem. An initial study of the microbial composition of these systems could also be used as a baseline and future indicator of the success of conservation and restoration efforts in these areas. We therefore set out to study the microbial composition of these land-locked saltmarshes that are a relic of ancient oceans that once covered the middle of North America.

## 2. Materials and Methods

### 2.1. Environmental Sampling

We collected samples from three areas near the crossing of Small Salt Creek and Salt Creek near Lincoln, NE, in early October 2020 (Lat. 40°52′54.42″ N; Lon. 96°39′35.82″ W) (Figure 1A). Two samples were taken from a larger pond area (Figure 1B: SM2B and SM2C) approximately 6 m apart, while two others were taken from nearby puddles with a visibly different ecosystem: SM2A showed high levels of insect activity and plant decay, while SM2E had visible indications of white sulfur deposits (Figure 1). All sampling sites had a salinity of >38 PPT (measured with a handheld salinity hydrometer (Fluvial Sea)), nitrate levels of ~5 mg/L (measured with the Nitrate Freshwater and Saltwater Test Kit from API), but differed in pH values: SM2B/C pH 9–9.5; SM2E pH 8.0, and SM2A pH 6.3. Samples were collected in sterile collection tubes and immediately transferred to the lab where they were stored at 4 °C the same day. The next day, 8 mL of each sample were centrifuged for 15 min at 16,000× *g* to form a biomass pellet.

### 2.2. Nucleic Acid Extraction and 16S rRNA Amplicon Sequencing

Total DNA was extracted using the PureLink Microbiome DNA Purification Kit (Invitrogen). Utilizing Qubit and NanoDrop, we determined the quality and quantity of DNA, showing an absorbance ratio of 260/280 between 1.83 (SM2E) and 2.00 (SM2A). A 16S rRNA amplicon sequencing library was prepared for each sample, following the 16S Metagenomic Sequencing Library Preparation protocol (Illumina, San Diego, CA, USA). Amplicon primers targeting the V3 and V4 region [11], including the Illumina adapter overhang sequences, are described in the Illumina library prep protocol and were synthesized by Sigma Aldrich. The samples were sequenced using a 1.8 pM library with an Illumina MiniSeq. Paired-end (2 × 150 bp) sequencing generated 1,191,674 reads (SM2A), 1,298,748 reads (SM2B), 1,292,958 reads (SM2C), and 1,141,458 reads (SM2E).

### 2.3. Sequence Read Analysis

The primer sequences were removed, and reads with low quality scores (average score < 20) were filtered out using the FASTQ Toolkit within BaseSpace (Illumina, Version 2.2.0). The 16S Metagenomics app within BaseSpace (Version 1.0.1) was used to perform a taxonomic classification, using an Illumina-curated taxonomic database RefSeq RDP 16S v3 [12] and the RDP naive Bayes taxonomic classification algorithm with an accuracy of >98.2% at the species level [13]. Default parameters were used for all software unless otherwise noted.

### 2.4. Enrichment Cultivation Strategy

We used RCVB media, which contains DL-malic acid as the organic carbon source, for the enrichment of photosynthetic bacteria in the SM2B salt marsh sample [14]. The NaCl concentration in the media was increased to 35 g/L to reflect the salinity of the sampling sites. After autoclaving, the RCVB media was supplemented with niacin (1 mg/L), thiamine (1 mg/L), vitamin B12 (10 mg/L), and sodium sulfide (0.03% Na_2_S.9H_2_0), and the pH was adjusted to pH 9.0. Five milliliters of the original sample were used to inoculate 45 mL of media in a 50 mL falcon tube and sealed off tightly. The tubes were placed at room temperature in front of a window for several weeks. This sample was labeled SM_Orange-Green, and two milliliters were used for genomic DNA extraction.

### 2.5. Whole-Genome Sequencing

A 2 mL sample of the enrichment cultures (SME_Orange-Green) was precipitated by centrifugation (10 min at 5000× *g*). DNA was isolated from these pellets using the GeneJet DNA purification kit (Thermo Scientific). In the case of the pink SM2E fraction that grew in the original isolation tube (SME_Pink), cells were scraped off from the tube wall using a sterile loop and resuspended in 180 µL of the GeneJet lysis buffer. Purified DNA samples were analyzed for purity and concentration using a Nanodrop and Qubit, showing an absorbance ratio of 260/280 of 1.85 (SME_Orange-Green) and 1.77 (SME_Pink).

The DNA libraries were prepared with the Nextera DNA Flex Library Prep Kit (Illumina). All genomes were sequenced using 500 µL of a 1.8 pM library, with an Illumina MiniSeq instrument, using paired-end sequencing (2 × 150 bp). Quality control of the reads was performed using FASTQC within BaseSpace (Illumina, Version 1.0.0), using a k-mer size of 5 and contamination filtering.

### 2.6. 16S rRNA Amplification

Degenerate primers were used for the 16S rRNA PCR reaction, described by Weisberg et al. [15], for the initial identification of the pink-enriched SM2E fraction (SME_Pink). The FD1 forward and the RP1 reverse primers were used. Amplified fragments were gel purified using the PureLink Quick Gel Extraction & PCR purification kit (Invitrogen) and sequenced by Sanger sequencing using the respective FD1 and RP1 sequencing primers (at the Iowa State University DNA core facility). The degenerate primers were designed to amplify most bacterial 16S rRNA, and given the known impurities in the SM2E sample, this could result in the amplification of 16S rRNA from different species; however, both of the sequencing reactions gave the same single result. Forward and reverse sequences were assembled (using a CLUSTALW alignment of the sequenced fragments) into one 16S rRNA sequence of 718 bp.

### 2.7. Metagenomic Binning Analysis

The sequencing reads of SME_Orange-Green and SME_Pink were used to perform a metagenomic binning using the Metagenomic Binning service within PATRIC [16]. Paired-end reads were used as the input, and default parameters were used. Sets of contig bins were constructed, with hits against contigs that have less than fourfold coverage or are less than 400 bp in length being removed. The contig pool was split into bins using reference genomes. Quality control of each bin was performed using checkM [17]. Each bin was automatically annotated using RAST within PATRIC [16], and consistency checks of the annotation were performed, producing a coarse score (percentage of roles that are correctly present or absent) and a fine score (percentage of roles that are correctly absent or present in the correct number). Identified genomes were ranked based on their coarse score, fine score, and completeness.

### 2.8. Whole-Genome Comparison

Average percentage nucleotide identity (ANIb) between the whole genomes was calculated using JSpecies [18]. A whole-genome-based phylogenetic tree was generated using the CodonTree method within PATRIC [16], which used PGFams as homology groups. For *Prosthecochloris*, 648 PGFams were found among these selected genomes using the CodonTree analysis, while 698 were found in the case of *Marichromatium*. The aligned proteins and coding DNA from single-copy genes were used for RAxML analysis [19,20]. iTOL was used for tree visualization [21].

## 3. Results and Discussion

### 3.1. Metagenomic Analysis: Taxonomic Overview

**Figure 2:**
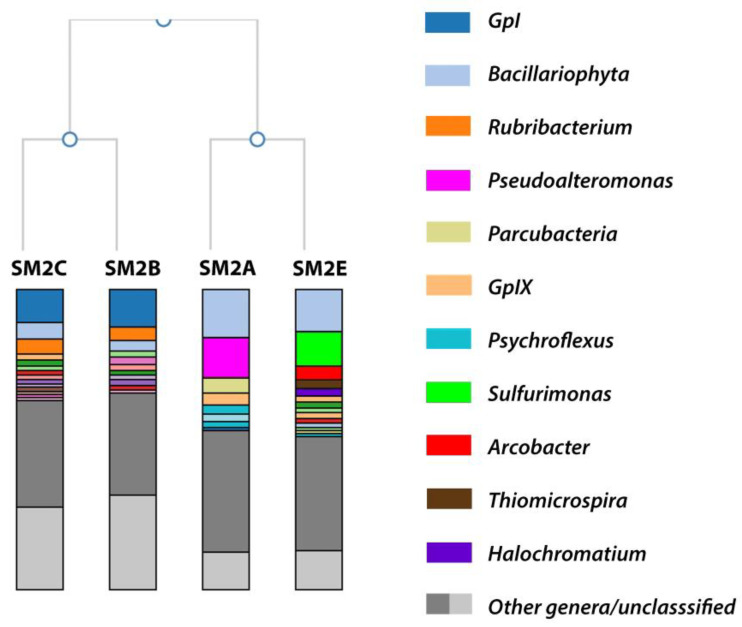
A dendrogram showing the hierarchical clustering of the metagenomes from the three sampling locations, based on genus-level classifications of 16S rRNA gene amplicon samples. Only families with >3% of the sequencing reads are represented in the figure legend.

According to a Shannon species diversity analysis (run within the 16S Metagenomics analysis) [22,23], each of the samples contained the following total potential species hits and Shannon diversity index (SDi): SM2A 2495 species (SDi 2.92); SM2B 1470 species (SDi 2.39); SM2C 1843 species (SDi 2.73), and SM3E 2646 (SDi 3.20). An aggregate comparison and hierarchal clustering of the taxonomic classification results of all four samples was performed within the 16S Metagenomic analysis (Figure 2). Samples SM2A and SM2E had more than 87% of the reads identified at the genus level, while sample SM2B and SM2C had ~70% of the reads identified at the genus level.

Table 1 provides an overview of the relative abundance (as a percentage of the total sequencing reads for each sample) of the most abundant genera from Figure 2. It was clear that a large portion of the species identified in all of the samples belonged to diatomic algae (*Bacillariophyta*, identified based on their chloroplast 16S rRNA) or cyanobacteria (GpI and GpIX), which were likely responsible for the observed green color of the sampling sites. However, the samples from the different locations showed substantial differences in the rest of the microbiome composition. At the phylum level, the majority of bacteria in each sample belonged to the *Proteobacteria*: SM2A 41.3%; SM2B 26.2%; SM2C 28.2%; and SM2E 51.3% of total reads. However, the phylum in SM2A was mainly composed of *Gammaproteobacteria* (28.7% of total reads) and *Alphaproteobacteria* (6.4%), while SM2B and SM2C had more *Alphaproteobacteria* (20.7 and 19.7%) and less *Gammaproteobacteria* (4.1 and 5.6%). SM2E on the other hand mainly contained *Epsilonproteobacteria* (23.0%) and *Gammaproteobacteria* (18.0%).

#### 3.1.1. SM2B/C Analysis

The reads identified from the SM2B and SM2C samples were very similar and contained abundant representation of the bacterial genus *Rubribacterium* (~5% of total reads), the cyanobacterial genus *GpI* (13% of total reads), and the algal genus *Bacillariophyta* (5% of total reads) (Table 1). Cyanobacteria and algae are commonly found in marine or coastal salt marshes, so it was not surprising that these were well represented here. They were likely responsible for the green coloration of the sampling sites. *Bacillariophyta* are diatoms commonly found in oceans, waterways, and soils around the world [24,25]. Together with cyanobacteria, these species enrich the bacterial ecosystem with organic matter and nutrients through oxygenic photosynthesis [26,27]. *Rubribacterium* is an alkaliphilic purple nonsulfur bacterium, belonging to the family of *Rhodobacteraceae*. The single species of this genus has only been isolated from an Eastern Siberian soda lake [28]. Purple nonsulfur bacteria have been found in weakly and moderately mineralized soda lakes [29,30,31]; however, only three alkaliphilic species are known. Two belong to the genus *Rhodobaca* [32,33], and one is *Rubribacterium polymorphum* [28]. It was intriguing that this rare *Rubribacterium* had the highest bacterial representation in the Nebraska Salt Marsh sample. Even though the two locations where *Rubribacterium* was found are several thousands of miles apart, there are certain similarities between the isolation sites. The Eastern Siberia soda lake had a pH of 9.5, identical to the pH of the SM2B/C samples. Both locations had a high mineralization of the water (22 g/L of the Eastern Siberia lake vs. 38 g/L for the Nebraska Salt Marsh samples), and in both locations, the water had high amounts of green algae and cyanobacteria ([28] and this study). *Rubribacterium* grows well in the dark on organic substrates with a pH optimum of 8.5–9.5 and an optimal NaCl concentration of 10 g/L, although it has been shown to grow up to 40 g/L [28].

#### 3.1.2. SM2A Analysis

Both SM2A and SM2E also contained a substantial representation of *Bacillariophyta* (~17% of total reads) and the cyanobacterial genus *GpI*X (4 and 2% of total reads, respectively) (Table 1). However, the bacterial representation was substantially different. SM2A mainly contained *Pseudoalteromonas* (14%), *Parcubacteria* (7.3%), and *Psychroflexus* (6.3%) and a minor fraction of *Salinivibrio* (2%) (Table 1 and Figure 2).

*Pseudoalteromonas* contains marine species that are found to be associated with higher plant and insect organisms, and several species prevent the fouling of aquatic environments with their bacteriolytic and algacidal activity [34]. This has been found to promote the survival of other marine organisms. *Parcubacteria* have been identified as symbiotic bacteria from a range of anoxic environments [35,36]. Members of this genus have limited mechanisms for energy and nutrient conservation (for example, lacking genes for the TCA cycle and electron transport), making them dependent on symbiosis for survival. They have been implicated in environmental hydrogen and sulfur cycling in anoxic environments [37]. The observation of both *Pseudoalteromonas* and *Parcubacteria* coincides well with the observed presence of higher insect activity and decaying plants in the SM2A sampling location (Figure 1B). *Psychroflexus* is a psychrophilic genus belonging to the *Flavobacteriaceae* and has been isolated from Antarctic sea ice [38], hypersaline lakes and sea basins [39,40,41], and the microbial consortium on the surface of Austrian cheese [42]. Similarly, *Salinivibrio* consists of halophilic bacteria commonly found in hypersaline aquatic habitats and salted foods [43,44]. Finding these species is consistent with the cold, saline environment of the Nebraska Salt Marshes in the fall.

#### 3.1.3. SM2E Analysis

SM2E on the other hand contained a substantial bacterial representation of *Sulfurimonas* (16.3% of total reads) and smaller fractions of *Arcobacter* (5%), *Thiomicrospira* (3.5%), and *Halochromatium* (2%) (Table 1 and Figure 2). *Sulfurimonas* species have been identified in distinct environments such as hydrothermal deep-sea vents, marine sediment, and coastal estuaries [45,46,47]. *Sulfurimonas* are sulfur-oxidizing bacteria, and it has been shown recently that *Sulfurimonas* species play an important role in coastal mangrove environments for the oxidation of the toxic sulfide produced by sulfur-reducing bacteria, like *Sulfurospirillum* [46,47].

*Arcobacter* are *Campylobacter*-like organisms that have been found in both animal and environmental sources, but compared to *Campylobacter*, they can grow at lower temperatures and are aerotolerant. Arcobacter has been isolated from a wider range of environments. One halophilic species, *A. halophilus,* has been collected from a hypersaline lagoon in Hawaii [48]. *A. nitrofigilis* is a nitrogen-fixing bacterium isolated from the roots of the salt marsh plant *Spartina alterniflora* [49,50]. Based on a provisional identification, it appears that the dominant plant species in the NE Salt Marsh sampling area is the invasive *Phragmites* sp. Further studies will be necessary to determine whether or not this *Arcobacter* species is also important for nitrogen fixation of *Phragmites* sp., similar to what was found for the salt marsh *Spartina alterniflora* [49,50]. *Arcobacter sulfidicus* is an obligate microaerophile that oxidizes sulfides and is a producer of filamentous sulfur [51]. Large populations of this bacterium produce mats of this solid, white sulfur filament [52]. This is consistent with the observed white sulfur deposits at the SM2E sampling site (Figure 1B).

*Thiomicrospira* is another colorless sulfur bacterium that derives energy from the oxidation of reduced sulfur compounds. The genus *Thiomicrospira* contains sixteen species [53]. Many of these, for example *Tms. aerophila*, *Tms. microaerophila*, *Tms. cyclica*, and *Tms. sibirica*, have been isolated from alkaline environments: Mono Lake (U.S.), Soap Lake (U.S.), and a soda lake in northern Russia [54,55,56]. The fact that these are obligatory alkalophilic and obligately chemolithoautotrophic sulfur-oxidizing bacteria is consistent with the alkaline conditions of the NE Salt Marsh area.

*Halochromatium* bacteria occur in hypersaline habitats. They belong to the *Chromatiaceae*, which is the main family of the purple sulfur bacteria [57,58]. Purple sulfur bacteria use reduced sulfur (e.g., sulfide or thiosulfate) as an electron donor in their photosynthetic pathways, thereby often producing granules of elemental sulfur [57,58]. After about two weeks of storing the SM2E sample at 4 °C, a significant pink growth occurred on the wall of the original sampling tube. A sample was taken from the pink substrate for genomic DNA extraction and used for 16S rRNA PCR and genome sequencing. The result showed that this was a species of *Halochromatium*, most closely related to *Halochromatium roseum* JA134, based on the 16S rRNA comparison (NCBI BLAST identity 99%; 710/718 bp). The whole genome was only partially completed and (as expected) showed significant contamination (>1000 contigs, estimated 40% contamination), but metagenomic binning further confirmed that this species belonged to the *Chromatiaceae*.

The heavy presence of sulfur-oxidizing and filamentous sulfur-producing species in the SM2E sample was consistent with the visible white sulfur precipitates in that sample area. There appeared to be a low abundance of sulfur-reducing species, for example *Sulfurospirillum* was found in 0.38% of total reads, while only two genera of *Desulfobacteraceaea* (*Desulfonema* and *Desulfotignum*) were found at 0.65 and 1.0%, respectively. This low abundance and limited plant growth in that area suggested that the homeostasis in the SM2E sampling area was possibly disrupted and an excess of oxidized sulfur components was being produced. The SM2E area appears to have been formed after the main salt marsh pond retracted, possibly due to seasonal fluctuations.

### 3.2. Genomic Analysis of Enrichment Cultures

Since the 16S rRNA amplicon metagenomic data showed that many of the bacterial representations were found to be from less characterized or rare photosynthetic bacteria, we decided to attempt an enrichment in order to obtain whole-genome or partial-genome data that could be used for a better identification and taxonomic classification of these species.

SM2B was used to inoculate an alkaline (pH 9.0), saline, sulfide-containing medium for photosynthetic sulfur bacteria cultivation. After about two weeks of anaerobic incubation with a night-dark light cycle, the culture showed a faint orange-brown color, which turned dark green in the following two weeks. A two milliliter sample of this (SM_Orange-Green) was used for genomic DNA extraction and genome sequencing. Since we did not expect this to be a single pure culture, we used the sequencing reads to perform a metagenomic binning within PATRIC [16]. We obtained two valuable bins: one genome related to *Prosthecochloris* sp. (97.9% coarse consistency; 96.4% fine consistency), the other to *Marichromatium* sp. (96.4% coarse consistency; 92.3% fine consistency), both with 100% completeness. These strains were designated as *Prosthecochloris* sp. SM2 and *Marichromatium* sp. SM2, for “Salt Marsh Location 2”. We also obtained three bins containing partial, incomplete genomes, all three identified as *Sulfurospirillum* species. Although *Prosthecochloris* and *Marichromatium* were not found in high abundance in the 16S rRNA amplicon analysis (see above), both species were found to be present in the SM2B sample. *Prosthecochloris* was found be present in 1.7% of the total reads in sample SM2B, while *Marichromatium* was found to be at 0.8%. However, 16S rRNA amplicon analysis limits resolution at the species and sometimes at the genus level since it only uses a fragment of the 16S rRNA gene for identification. The actual representation of these species might be higher since we found the family of *Chlorobiaceae* in 2.5% and the *Chromatiaceae* in 1.6% of the total reads. The anaerobic, high sulfide, and halophilic conditions of the enrichment cultivation were certainly favorable for these photosynthetic species.

#### 3.2.1. *Prosthecochloris* sp. SM2

**Figure 3:**
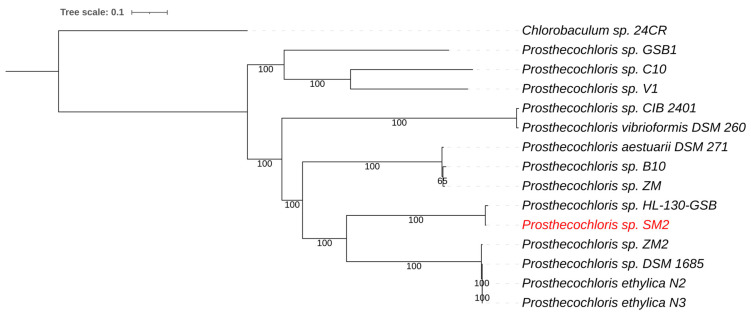
Whole-genome-based phylogenetic tree of all sequenced *Prosthecochloris* species. The phylogenetic tree was generated using the CodonTree method within PATRIC [16], which used PGFams as homology groups. The support values for the phylogenetic tree were generated using 100 rounds of the “Rapid bootstrapping” option of RaxML [19]. *Chlorobaculum* sp. 24CR was used as an outgroup [63]. The branch length tree scale is defined as the mean number of substitutions per site, which is the average across both nucleotide and amino acid changes. The NE Salt Marsh *Prosthecochloris* sp. SM2 is shown in red.

*Prosthecochloris* are green sulfur bacteria (*Chlorobiaceae*) that are anoxygenic phototrophic bacteria. The sulfur metabolism of green sulfur bacteria involves the oxidation of sulfide and the deposition of elemental sulfur globules outside the cells [59]. The *Prosthecochloris* genome was assembled into 112 contigs with a total genome size of 2.43 Mb and a G+C content of 51.8%. Average nucleotide identity (ANIb) comparison showed that the genome sequence of the NE Salt Marsh *Prosthecochloris* was most similar to strain HL130-GSB, with an average nucleotide identity (ANI) of 98.4%. This ANI value was above 95%, which is the arbitrary cutoff value for species differentiation [18], indicating that these strains likely belonged to the same species. A whole genome-based phylogenetic tree for *Prosthecochloris* placed the green component as the closest relative to strain HL-130-GSB (Figure 3). This was consistent with the ANI comparisons mentioned above. *Prosthecochloris* HL-130-GSB was isolated from a cyanobacterial mat obtained from Hot Lake, a saline, high-sulfate, meromictic lake in WA, USA [60].

Not surprisingly, the *Prosthecochloris* sp. SM2 genome contains homologues of the genes for sulfur oxidation, SoxB and SoxYZ, the latter of which is genetically clustered with a flavocytochrome c:sulfide dehydrogenase encoding gene. This indicates that *Prosthecochloris* sp. SM2 is indeed capable of sulfur oxidation. We recently showed that several *Prosthecochloris* strains that form syntrophic interactions with sulfur-reducing bacteria contain very large agglutination proteins [61]. These agglutination proteins function as excreted adhesion proteins that are important for the formation of syntrophic interactions and larger bacterial consortia [61,62]. The formation of these interactions aids in the mutual exchange of metabolites. At this point, it is not known if this new strain also forms syntrophic complexes; however, we were able to identify the same large agglutination proteins: a structural toxin protein RtxA homologue and a large outer membrane adhesion protein (“tandem-95 repeat” protein), followed by a smaller gene encoding an agglutination protein (TolC family type I secretion outer membrane protein), an ABC transmembrane transporter (type I secretion system ATPase), and a HlyD homologous protein (type I secretion membrane fusion protein). This indicates that this new *Prosthecochloris* sp. SM2 is also capable of producing the adhesion complex that has been found to be important for the formation of close interspecies interactions.

#### 3.2.2. *Marichromatium* sp. SM2

**Figure 4:**
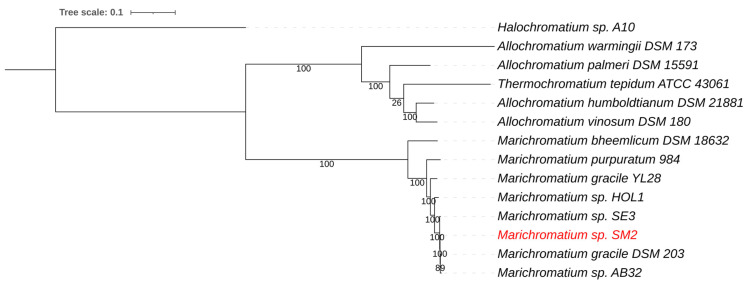
Whole genome-based phylogenetic tree of sequenced *Chromatiaceae* species. The phylogenetic tree was generated using the CodonTree method within PATRIC [16], which used PGFams as homology groups. The support values for the phylogenetic tree were generated using 100 rounds of the “Rapid bootstrapping” option of RaxML [19]. *Halochromatium* sp. A10 was used as an outgroup. The branch length tree scale is defined as the mean number of substitutions per site, which is the average across both nucleotide and amino acid changes. The NE Salt Marsh *Marichromatium* sp. SM2 is shown in red.

*Marichromatium* is a small genus belonging to the purple sulfur bacteria (*Chromatiaceae*) [57]. It was found that the rRNAs of *Chromatiaceae* form two major lines of descent, one composed primarily of halophilic and the other of mainly fresh-water origin. *Marichromatium* is placed within the halophilic branch of the *Chromatiaceae* [57]. Several strains of *Marichromatium* are known, and the genus appears to be commonly found throughout the world; however, there does not seem to be much variation in the group as a whole. The *Marichromatium* genome from the enriched NE Salt Marsh sample was assembled into 293 contigs with a total genome size of 3.94 Mb and a G+C content of 67.6%. Average nucleotide identity (ANIb) comparison showed that the genome sequence was most similar to *Marichromatium gracile* DSM203^T^, with an average nucleotide identity (ANI) of 98.4%. A whole genome-based phylogenetic tree for *Marichromatium*, including *Halochromatium*, *Allochromatium,* and *Thermochromatium* genomes, placed the green component as the closest relative to *Marichromatium gracile* (Figure 4). *Marichromatium gracile* DSM203^T^ was isolated form marine waters at Headley Harbour, Massachusetts, USA [64].

As expected, the *Marichromatium* sp. SM2 genome also contains the SoxB and SoxYZ genes for sulfur oxidation. It has recently been shown that the halophilic *Marichromatium* species will use glycine betaine as an osmotic adaptation to a high-salt environment [64]. A feature search of the genome within PATRIC showed that this genome contains several genes for glycine betaine uptake and metabolism, including a betaine aldehyde dehydrogenase (EC 1.2.1.8), a glycine betaine transporter OpuD, a secondary glycine betaine transporter BetU, and a glycine betaine ABC transporter (consisting of the substrate-binding protein OtaC, permease protein OtaB, and the ATP-binding protein OtaA). This suggests that this species is well adapted to the high salt environment of the NE Salt Marsh.

#### 3.2.3. *Sulfurospirillum*

*Sulfurospirillum* are sulfur-reducing, nitrogen-fixing bacteria that have been found to be important for hydrogen and sulfur-based syntrophic interactions in several marine environments [47,65,66,67]. Sulfur-reducing bacteria like *Sulfurospirillum* produce sulfide that can be toxic to plants and other oxygenic photosynthetic species like algae or cyanobacteria. They are typically found to work in balance with sulfur-oxidizing or sulfur-producing species [47,66]. The genome fragments we obtained from metagenomic binning were only partially complete (22 to 93 percent completeness) with varying levels of contamination present (6% to 61%). This made an accurate species annotation impossible; however, strains UBA2217, SL2-2, and UBA12182, were found to be the closest relatives based on metagenomic analysis. All three were assembled genomes from metagenomic samples from an oil production facility and contaminated mine tailings [68] or a sludge bioreactor to treat contaminated water [69].

*Sulfurospirillum* are the only organohalide-respiring *Epsilonproteobacteria* described so far, and they grow on many toxic compounds, allowing them to thrive in polluted habitats [65]. Electron acceptors for anaerobic respiration used by all *Sulfurospirillum* species so far are nitrate, fumarate, and sulfur (including polysulfide). Interestingly, none of the species reduce sulfate in their energy metabolism, so the species are involved in their environment as sulfur reducers. Although several marine species have been found, only one alkaliphilic species has been isolated, *Sulfurospirillum alkalitolerans* [70]. This species was isolated from a lab bioreactor that was used to remove H_2_S from waste gasses. The species shows a pH range from 7.1–9.7 and salt tolerance up to 1.5 M NaCl. From our enrichment analysis, it appeared that *Sulfurospirillum* was the dominant sulfur reducer in the high-saline, alkaline environment of these NE Salt Marshes. Since both *Prosthecochloris* and *Sulfurospirillum* previously have been found to be involved in syntrophic interactions with other species [61,67], it is possible that the sulfur-producing *Prosthecochloris* sp. SM2 forms a similar syntrophy, and close contact interactions, with the *Sulfurospirillum* in our enrichment cultures, although further physiological and biochemical analysis will be required to investigate this further.

### 3.3. The Salt Marsh Sulfur Cycle

**Figure 5:**
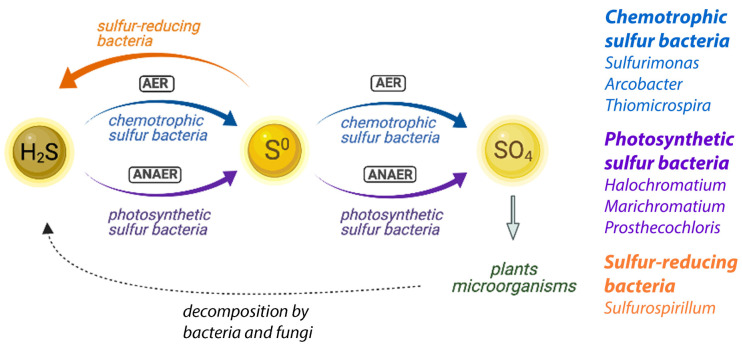
Simplified overview of the proposed sulfur cycling in microbial ecosystems. Bacterial genera identified in the NE Salt Marsh samples that are assumed to be involved are indicated for each group. AER: aerobic reactions; ANAER: anaerobic reactions. Image created using BioRender.com.

The sulfur cycle and its microbial components have been extensively studied in microbial terrestrial and marine ecosystems [8,9,71]. A simplified model is summarized in Figure 5, where we highlight the different bacterial groups assumed to be involved in the context of the NE Salt Marsh ecosystem.

It has been shown previously that marine microbial benthic ecosystems are dominated by essentially a few functional groups of microbes [8,9], which makes them an ideal model system to study the cycling of sulfur and organic matter. Sulfur is an essential element in all organisms; however, due to the large variety of the possible oxidation states of sulfur, between −2 (as in sulfide) and +6 (as in sulfate), different sulfur species can be useful as electron acceptors or electron donors in a variety of different processes. The processes of sulfur cycling are predominantly mediated by bacteria, and their role in the sulfur cycle is therefore crucially important to maintain an ecosystem.

In simplified terms, the microbial ecosystems consist of cyanobacteria and algae that perform aerobic photosynthesis, which causes oxygen depletion and provides growth substrates for other species. As a consequence of lysis, excretion, and fermentation, organic compounds are produced that are partially or completely degraded by a variety of bacteria and fungi. Some of those species are sulfate-reducing bacteria that produce sulfide, through the reduction of sulfates. Sulfur-reducing bacteria, like *Sulfurospirillum*, will contribute to the sulfide (H_2_S) production by further reduction of sulfur (or polysulfides) (orange pathway in Figure 5). This sulfide is reoxidized to sulfate by purple photosynthetic bacteria and colorless sulfur bacteria (purple and blue pathways in Figure 5). Sulfide is inhibitory to oxygenic phototrophs, like cyanobacteria and algae, and its removal by the anaerobic purple and colorless sulfur bacteria is crucial to balance the sulfur cycling in an ecosystem. In addition, these sulfur bacteria produce reducing equivalents for CO_2_ reduction. Green sulfur bacteria are obligatory anaerobes and grow best under low light conditions, and if they are present in these systems, they are typically found underneath the purple sulfur bacteria [72,73]. They contribute to the anaerobic sulfide oxidation pathway (purple pathway in Figure 5). Purple nonsulfur bacteria, like *Rubribacterium*, also contribute by anoxygenic photosynthesis and typically use hydrogen as an electron donor, but they can sometimes also use sulfide (at a lower concentration than the photosynthetic sulfur bacteria). We identified several representatives of each of these groups in our microbial analysis of the NE Salt Marshes, and it is clear that a combined, consorted action of all these groups is needed to maintain a balance of nutrient cycling in the ecosystem (Figure 5).

## 4. Conclusions

The various metabolic capacities of the NE Salt Marsh microbial community are responsible for the biogeochemical cycling of important chemical elements like sulfur, nitrogen, and carbon. From studies of coastal salt marshes, marine microbial sediments, and also ancient stromatolites, it appears that these microbial ecosystems have been resilient for billions of years [9,71]. It is therefore important to study and attempt to preserve these locally important ecosystems. If nothing else, they provide an important glimpse into the biochemical balancing of the planet, now and in the past. The NE Salt Marsh microbiome consists of alkalophilic and halophilic species that appear to be well adapted to this specialized environment. The microbial ecosystem appears to be well balanced as far as sulfur and other nutrients; nevertheless, urban development, agricultural runoff, and the growth of invasive plant species are some of the current threats to maintaining these vital ecosystems. Restoration and conservation efforts of native ecosystems for the most part focus on the insect and plant community; however, a recent study by Lynum et al. [74], specifically on passive salt marsh restoration, showed that the underlying microbial community’s conservation is crucial and paramount to any preservation of marsh vegetation. Although the microbial community can respond quickly in a positive direction, the process of microbial restoration can take several years, before any return to native marsh vegetation is visible [74]. With the current threats to this inland salt marsh system in Nebraska, it is important to understand the underlying microbial composition and complex biochemistry. This current study establishes a good baseline for further studies on how microbiological diversity, nutrient cycling, and bacterial ecology impact this locally important watershed.

## Figures and Tables

**Table 1:** Relative abundance at the genus level of the most abundant genera in each of the four samples. The abundance is given as a percentage of the total sequencing reads for each sample, as calculated by the 16S Metagenomics analysis in BaseSpace (Illumina). Abundances over 2% are indicated in bold and discussed in the text; n.d.: not detected.

Classification	SM2A	SM2B	SM2C	SM2E
*GpI*/*GpIX*	**3.9**	**13.9**	**12.3**	**2.3**
*Bacillariophyta*	**17.1**	**3.4**	**6.4**	**16.8**
*Rubribacterium*	<2	**4.8**	**5.0**	<2
*Pseudoalteromonas*	**14.1**	n.d.	n.d.	<2
*Parcubacteria*	**7.3**	n.d.	<2	<1
*Psychroflexus*	**6.3**	<1	<1	<2
*Sulfurimonas*	<2	<2	<2	**16.3**
*Arcobacter*	<2	n.d.	n.d.	**5**
*Thiomicrospira*	<2	<2	<1	**3.5**
*Halochromatium*	<1	<2	<2	**2.1**
*Salinivibrio*	**2.1**	n.d.	<1	<1
Unclassified	12.4	31.4	27.3	13

## Data Availability

The 16S rRNA gene amplicon data sets and WGS have been deposited at DDBJ/ENA/GenBank under Project PRJNA714941. The 16S rRNA gene amplicon data sets can be accessed with SRA Accession Numbers SRR13980338 (SM2A), SRR13982897 (SM2B), SRR13981552 (SM2C), and SRR13981842 (SM2E).
